# Decoding Single and Paired Phonemes Using 7T Functional MRI

**DOI:** 10.1007/s10548-024-01034-6

**Published:** 2024-01-23

**Authors:** Maria Araújo Vitória, Francisco Guerreiro Fernandes, Max van den Boom, Nick Ramsey, Mathijs Raemaekers

**Affiliations:** 1 Brain Center Rudolf Magnus, Department of Neurology and Neurosurgery, University Medical Center Utrecht, Utrecht, The Netherlands; 2 Department of Physiology and Biomedical Engineering, Mayo Clinic, Rochester, MN USA

**Keywords:** Functional Magnetic Resonance Imaging; Speech brain-computer Interface, Speech Neuroprosthesis, Speech Production, Phonemes, Sensorimotor

## Abstract

Several studies have shown that mouth movements related to the pronunciation of individual phonemes are represented in the sensorimotor cortex. This would theoretically allow for brain computer interfaces that are capable of decoding continuous speech by training classifiers based on the activity in the sensorimotor cortex related to the production of individual phonemes. To address this, we investigated the decodability of trials with individual and paired phonemes (pronounced consecutively with one second interval) using activity in the sensorimotor cortex. Fifteen participants pronounced 3 different phonemes and 3 combinations of two of the same phonemes in a 7T functional MRI experiment. We confirmed that support vector machine (SVM) classification of single and paired phonemes was possible. Importantly, by combining classifiers trained on single phonemes, we were able to classify paired phonemes with an accuracy of 53% (33% chance level), demonstrating that activity of isolated phonemes is present and distinguishable in combined phonemes. A SVM searchlight analysis showed that the phoneme representations are widely distributed in the ventral sensorimotor cortex. These findings provide insights about the neural representations of single and paired phonemes. Furthermore, it supports the notion that speech BCI may be feasible based on machine learning algorithms trained on individual phonemes using intracranial electrode grids.

## Introduction

Throughout the past decades the field of brain-computer interfaces (BCI) has undergone unprecedented advancements. One major goal of BCI research has been the development of communication devices. Whereas healthy people are able to communicate verbally and non-verbally in order to interact with their environment, people who suffer from locked-in syndrome (LIS) have lost this ability. This syndrome is characterized by a loss of motor function, while consciousness and cognition remain intact. Research on the development of a BCI for communication has the potential to give these patients a “voice” again.

Many of the current BCI’s solutions are based on activity related to the execution of hand movements. Previous studies using intracortical microelectrode arrays translated neural signals into point-and-click commands to control a computer (Pandarinath et al. 2017; Nuyujukian et al. 2018). Furthermore, attempted handwriting movements were translated to text in real time (Willett et al. 2021). Other research discriminated between four complex hand gestures from American sign language alphabet from primary motor and sensory cortex using electrocorticography (ECoG) (Bleichner et al. 2016; Branco et al. 2017). One ECoG-BCI already enables patients with electrodes that are implanted on the hand motor area of the cortex to communicate at home with attempted hand movements (Vansteensel et al. 2016).

The use of motor activity related to speech production might represent an alternative, more intuitive target for a BCI implant. Neuronal networks related to speech production involve complex mechanisms that operate at several processing levels, where ultimately, the execution of coordinated and sequential movements of the articulators give rise to speech. While the decoding of words based on their pattern of articulator activity in the sensorimotor cortex should be possible in principle, the extensive vocabulary of any language makes this practically impossible. Instead, decoding might be performed on the phonemes that make up a word. Since the number of phonemes within a language is substantially lower, phonemes could serve to decode continuous speech (Mugler et al. 2014a, b; Wilson et al. 2020). Despite intracranial electrophysiology being the state of art for the development of naturalistic speech BCIs, fMRI can offer valuable neuroscientific insights than can be subsequently verified using intracranial methods. Previous studies with functional magnetic resonance imaging (fMRI) have successfully decoded articulator movements (Bleichner et al. 2015), consonant-vowel-consonant utterances (Correia et al. 2020), syllables (Otaka et al. 2008) and words (Grootswagers et al. 2013). Furthermore, invasive electrophysiological recordings allowed the classification of isolated phonemes (Blakely et al. 2008; Ramsey et al. 2018), single phonemes within words from the ventral sensorimotor cortex (Mugler et al. 2014a, b) and single phonemes within continuous speech from the dorsal sensorimotor cortex (Wilson et al. 2020). These studies provide evidence that articulators and phonemes are represented in the sensorimotor cortex during speech production and that it should be possible to develop a BCI system for continuous speech based on phonemes.

In this study we assess the decodability of single phonemes and combination of phonemes based on Blood-oxygen-level-dependent (BOLD) responses in the sensorimotor cortex using 7-Tesla fMRI. Importantly, we assess whether phonemes can be distinguished in isolation and within combinations, the latter being relevant for (eventually) detecting phonemes within spoken words, which would validate the prospect of reconstructing words based on classifiers for individual phonemes. In addition, we explored in more detail the topographical presentations such as laterality and presence in the adjacent non-motor language area (pars triangularis and pars opercularis). Finally, we explore differences in the spatial properties of neuronal representations between phonemes.

## Methods

### Participants

Fifteen subjects (average age 23.07 ± 2.54 years; 8 male) participated in the study. The study was approved by the medical-ethical committee of the University Medical Center Utrecht and all subjects gave their written informed consent in agreement with the Declaration of Helsinki (World Medical Association, 2013).

### Scan Protocol

The fMRI measurements were performed on a whole-body 7 Tesla MR system (Achieva, Philips Health Care, Best, Netherlands) using a 32-channel head-coil (Nova Medical, MA, USA). Subjects were provided with hearing protection while being in the scanner. Prior to the Phoneme task, a T_1_-weighted MP2RAGE (Marques et al. 2010) image of whole-brain was acquired. The functional data was recorded in transverse orientation using an EPI-sequence with the following parameters: repetition time (TR) = 1400 ms, echo time (TE) = 25 ms, flip angle (FA) = 60°, voxel size: 1.586 × 1.586 × 1.75 mm (Willett et al. 2021), 30 slices, ascending interleaved slice acquisition order, field of view (FOV) = 226.462 (AP) x 52.5 (FH) x 184 (LR) mm (Willett et al. 2021), anterior-posterior phase encoding direction and the slice stack was rotated so that the FOV covered the left pre- and postcentral gyrus. A total of 1680 functional images were acquired for each subject. At the start of functional imaging, a single functional image with identical parameters except for a reversed phase-encoding blip was acquired. Prism glasses allowed subjects to visualize the task displayed on a screen by a projection through a waveguide.

### Stimuli and task Design

For the experiment, participants overtly pronounced different phonemes (/t/, /p/, /ə/). These particular phonemes were chosen as their pronunciation primarily engages individual articulators, including tongue for /t/ (alveolar consonant), lip for /p/ (alveolar consonant), and larynx for /ə/ (central vowel). During the task, seven different stimulus classes were presented: three single phonemes (/t/, /p/, /ə/), three paired phonemes (/p/ /t/, /ə/ /t/, /p/ /ə/) and a single triplet phoneme (/ə/ /t/ /p/). The triplet phoneme class (/ə/ /t/ /p/) was excluded from the current analyses as we noticed that the discrimination of this condition was mainly driven by a higher number of phonemes that was being pronounced.

**Fig. 1:**
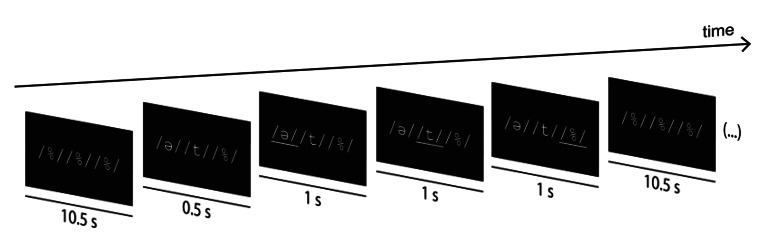
Schematic of the visual stimuli presented to participants during the fMRI task. The task included 6 functional runs, with each run consisting of 28 trials. Subjects were instructed to pronounce the underlined phoneme as shown in the displayed example

A slow event-related design was used for the task, with an inter-trial interval of 14s. The production of each individual phoneme was cued by a visual stimulus. For paired phoneme trials, there was an interval of 1s between the individual phoneme cues within the trial (Fig. 1). A /%/ was added to the visual stimuli to control for visual confounders in the trials with less than 3 phonemes. Participants were instructed to remain silent if /%/ was underlined. Each participant performed six runs of the task, where each run included four repetitions per class, providing a total of 24 trials per run. The trial order per run was randomly generated, with the same sequence being used for all participants (trials were perfectly balanced across participants). Prior to the scanning session, the participants were instructed on the proper pronunciation of the phonemes and the importance of restricting any other movements.

### Preprocessing

Preprocessing was performed using SPM12 (http://www.fil.ion.ucl.ac.uk/spm/), FreeSurfer 7.0 (Fischl 2012) (https://surfer.nmr.mgh.harvard.edu) and FSL 6.0 (Jenkinson et al. 2012) (https://fsl.fmrib.ox.ac.uk/fsl/fslwiki/FSL). The functional images were slice time corrected and realigned & unwarped using SPM12. Spatial distortions in the functional images were corrected using FSL’s topup in combination with the functional image that was acquired with reversed phase encoding blip (Andersson et al. 2003). The resulting functional images were coregistered to the anatomical image, the mean was subtracted from the timeseries for each run, and timeseries were high-pass-filtered using a kernel with a cut-off at 70 s to eliminate low frequency signal drifts. Subsequently, a cortical surface reconstruction was created based on the anatomical image using the FreeSurfer recon-all pipeline. Regions of interest were created based on the FreeSurfer parcellation according to the Desikan-Killiany atlas and included the sensorimotor cortex (precentral and postcentral gyrus), and pars triangularis and pars opercularis (referred to as PTPO, compromising Broca’s area for left hemisphere, and its homologue for the right hemisphere). An additional region of interest (ROI) was created that only included the cerebral white matter. This area was included as control area as it is assumed not to exhibit BOLD responses related to phoneme production. The white matter was slightly eroded to avoid inclusion of grey matter due to partial voluming effects.

### 2.1 Classification

Machine learning classification was performed on the functional data in the predefined ROIs using a MATLAB implementation of a multiclass Support Vector Machine (SVM) using a linear kernel (with a constraint parameter C = 1). SVM is known to be suitable for decoding patterns of fMRI activity involving high-dimensional data (Mitchell et al. 2004). We used the default regularization parameter since the number of voxels used was substantially higher than the number of trials, enabling a linear separability without the need to optimize the parameter C (Mourão-Miranda et al. 2006).

We used a leave-one-run out cross-validation scheme. Per subject, the 5 steps described here below were repeated for each of the 6 runs. With each iteration, one run was left out for training the model, and was subsequently used to test the model.

**Fig. 2:**
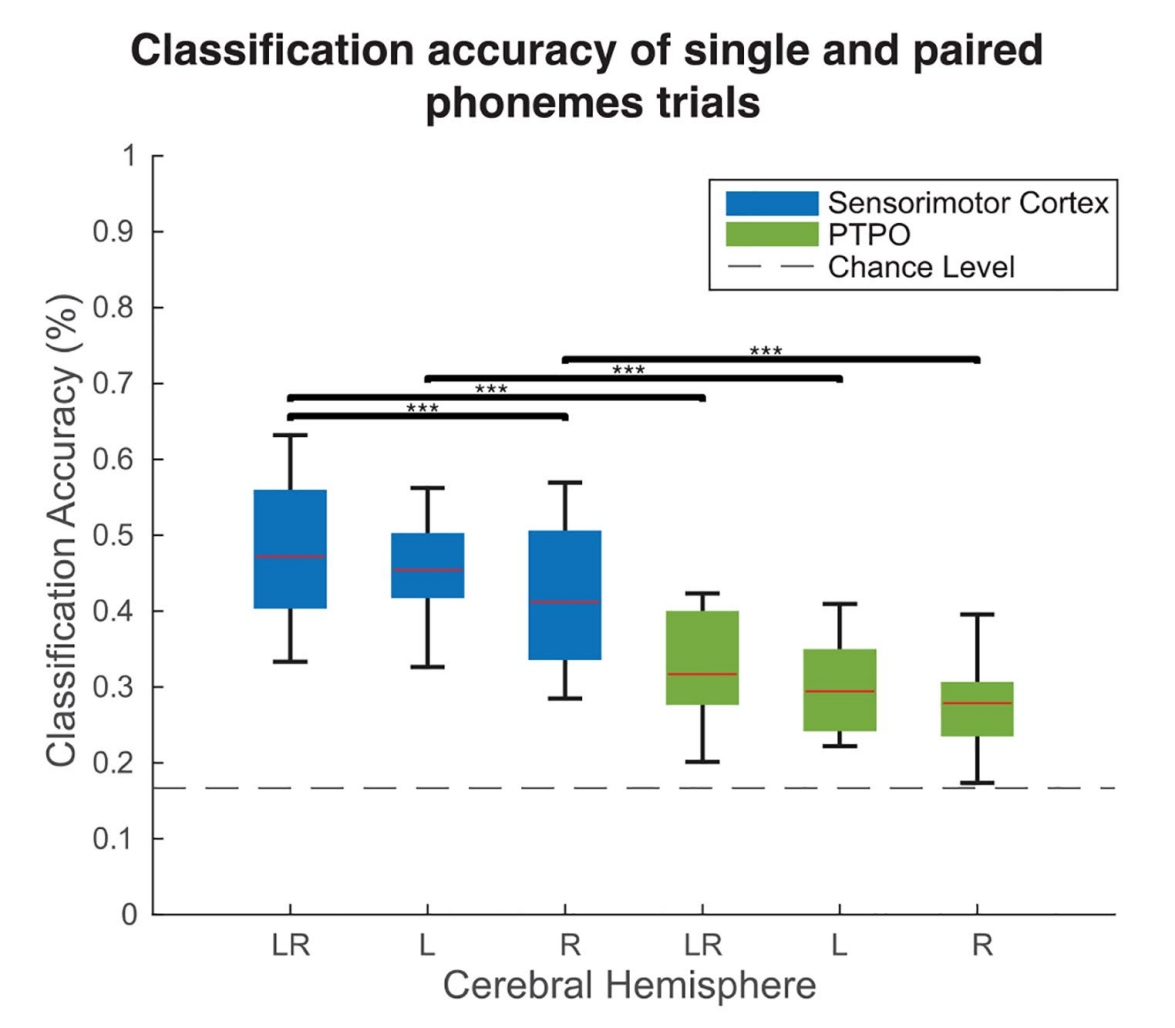
The group-mean (*n* = 15) BOLD signal (A) and classification accuracy (B) at each time point after stimulus onset. Three ROIs were used: sensorimotor cortex represented in blue, pars triangularis and pars opercularis in green and the cerebral white matter in yellow. The cerebral white matter was used as control, since a low BOLD signal and classification accuracy were expected. The shaded areas directly surrounding the curves represent the 95% confidence intervals. The grey shaded area marks the period during which participants were pronouncing the phonemes. The shaded red area marks the time interval used for the feature extraction (mean across images 4, 5 and 6). The baseline of responses in panel A is set at the signal amplitude during the last image, as it is least affected by motion artefacts or BOLD signal changes

1. A General Linear Model was fitted on the training data using one regressor per class plus six head movement parameters, providing a single t-map for each trial class;
2. A mask was created for each predefined ROI, including the 1000 voxels with the highest t-values across the six t-maps (the percentage of the total number of voxels per ROI this represents is displayed in Table 1);
3. From these voxels, the BOLD signal across images 4th, 5th or 6th (4.9–7.7 s) after each trial onset was averaged and extracted for the training and testing data for both isolated and paired phoneme trials (Bleichner et al. 2014, 2015; Bruurmijn et al. 2017). These images were chosen given they correspond to the peak of the BOLD response (Fig. 2). During these images no articulator movements that could cofound results were made;
4. The resulting values per voxel were used as features in training and testing the SVM;
5. Accuracies were calculated by classification of the trials in the test run. The resulting accuracies were averaged across these runs, and subsequently averaged across subjects.

This approach was repeated to classify all phoneme classes, only single phoneme, only paired phoneme trials, using single phonemes as training data and paired phonemes as testing data, using paired phonemes as training and single phonemes as testing data. For the classification with single phonemes, the data for training and testing included only trials with single phonemes. Similarly, classification with paired phonemes trials included only trials with paired phonemes for both training and testing. Additional analyses were performed to address possible confounding of classification results by motion artefacts due to phoneme production (see Results section). Furthermore, in order to assess if paired phonemes could be predicted based on the activity of isolated phonemes, the SVM was trained on activity during production of the single phoneme trials and used to classify paired phoneme trials. The opposite analysis was also performed, with the activity during paired phoneme trials being used to train the SVM, while testing the activity during single phoneme trials.

In order to establish a statistical significance (one sided, α = 0.05) threshold for the classification accuracies per subject, a Monte Carlo simulation was performed with 1000 permutations of the trial-class labels (Modarres and Good 1995; Nichols and Holmes 2002; Combrisson and Jerbi 2015). Group-level results were obtained by averaging classification accuracies and confusion matrices across subjects, and using *t*-tests for statistical inference. Bonferroni correction for multiple comparisons was used to account for fifteen comparisons: (1) sensorimotor cortex (bilaterally), (2) precentral gyrus (bilaterally), (3) postcentral gyrus (bilaterally), (4) pars triangularis and opercularis (bilaterally), (5) cerebral white matter (bilaterally), (6) sensorimotor cortex left hemisphere,7) precentral gyrus left hemisphere, 8) postcentral gyrus left hemisphere, 9) pars triangularis and opercularis left hemisphere, 10) cerebral white matter left hemisphere, 11) sensorimotor cortex right hemisphere,12) precentral gyrus right hemisphere, 13) postcentral gyrus right hemisphere, 14) pars triangularis and opercularis right hemisphere and 15) cerebral white matter right hemisphere. To assess whether there were significant differences between phoneme classes, a Friedman test was conducted followed by post-hoc pairwise comparisons with adjustment of the p-values to correct for multiple comparisons.

**Table 1:** Group means and standard deviations for the number and percentage of voxels for each ROI

		Number of voxels per ROI	Average percentage for each ROI
Sensorimotor Cortex	**Both Hemispheres**	7486 ± 1874	14 ± 3
	**Left Hemisphere**	3904 ± 1013	27 ± 6
	**Right Hemisphere**	3582 ± 918	30 ± 8
Pars triangularis and pars opercularis	**Both Hemispheres**	3924 ± 837	27 ± 9
	**Left Hemisphere**	1996 ± 374	52 ± 13
	**Right Hemisphere**	1928 ± 520	58 ± 30
Precentral Gyrus	**Both Hemispheres**	4266 ± 1117	25 ± 6
	**Left Hemisphere**	2203 ± 611	48 ± 12
	**Right Hemisphere**	2063 ± 538	52 ± 13
Postcentral Gyrus	**Both Hemispheres**	3220 ± 783	33 ± 8
	**Left Hemisphere**	1701 ± 421	62 ± 14
	**Right Hemisphere**	1519 ± 405	71 ± 19
Cerebral White Matter	**Both Hemispheres**	22,517 ± 4266	4.6 ± 0.9
	**Left Hemisphere**	11,259 ± 2130	37 ± 7
	**Right Hemisphere**	11,256 ± 2153	35 ± 6

### Univariate Results

To establish univariate (voxel-wise) results for the trials with one-phoneme, a GLM was created using a design matrix including factors for each condition and each run. The resulting volumes with beta-coefficients that represented single phoneme trials were averaged across runs, resulting in three volumes for each subject. These volumes were mapped to the surface using an enclosing voxel algorithm and while smoothing across the surface with a 12 mm gaussian kernel. The resulting surface-based activity maps were used as input for second-level analyses with one-sample and paired samples t-tests, comparing the single phoneme conditions against rest and against each other. Correction for multiple comparison was done using Random Field Theory.

### Surface-based Searchlight

A multivariate searchlight (Kriegeskorte et al. 2006) analysis was used to generate surface-based maps (Chen et al. 2011) indicating the presence of local information driving classification results. The volumetric functional data of each run was mapped to the cortical surface using FreeSurfer. An enclosing voxel (‘nearest neighbor’) algorithm was used to map voxel values to the vertices. The method for inclusion of vertices for each MVPA during the searchlight analysis was as follows:

1. All vertices were sequentially chosen as seed vertex.
2. All other vertices were sorted based on Euclidean distance to the seed-vertex in the inflated surface.
3. If multiple vertices were sampled from the same voxel, a single vertex was selected which was the one closest to the seed voxel.

For each vertex, a SVM was trained and tested including the activity in the 200 most proximate vertices (across the inflated surface) that were sampled from separate voxels. The 200 vertices closest to the seed vertex were selected as features for the MVPA. The SVM procedure was similar to the one described in the *Classification* section and resulted in a classification accuracy and confusion matrix for every vertex of the surface. For groupwise analysis, the single-subject results were registered to Freesurfer’s left/right symmetrical templates (*fsaverage-sym*) (Greve et al. 2013) and averaged across subjects. To correct for multiple comparisons, family wise error correction based on Random Field Theory was applied (*p* <.05).

## Results

### Single and Paired Phonemes can be Decoded Bilaterally from both the Sensorimotor Cortex and pars Triangularis and Opercularis

**Fig. 3:**
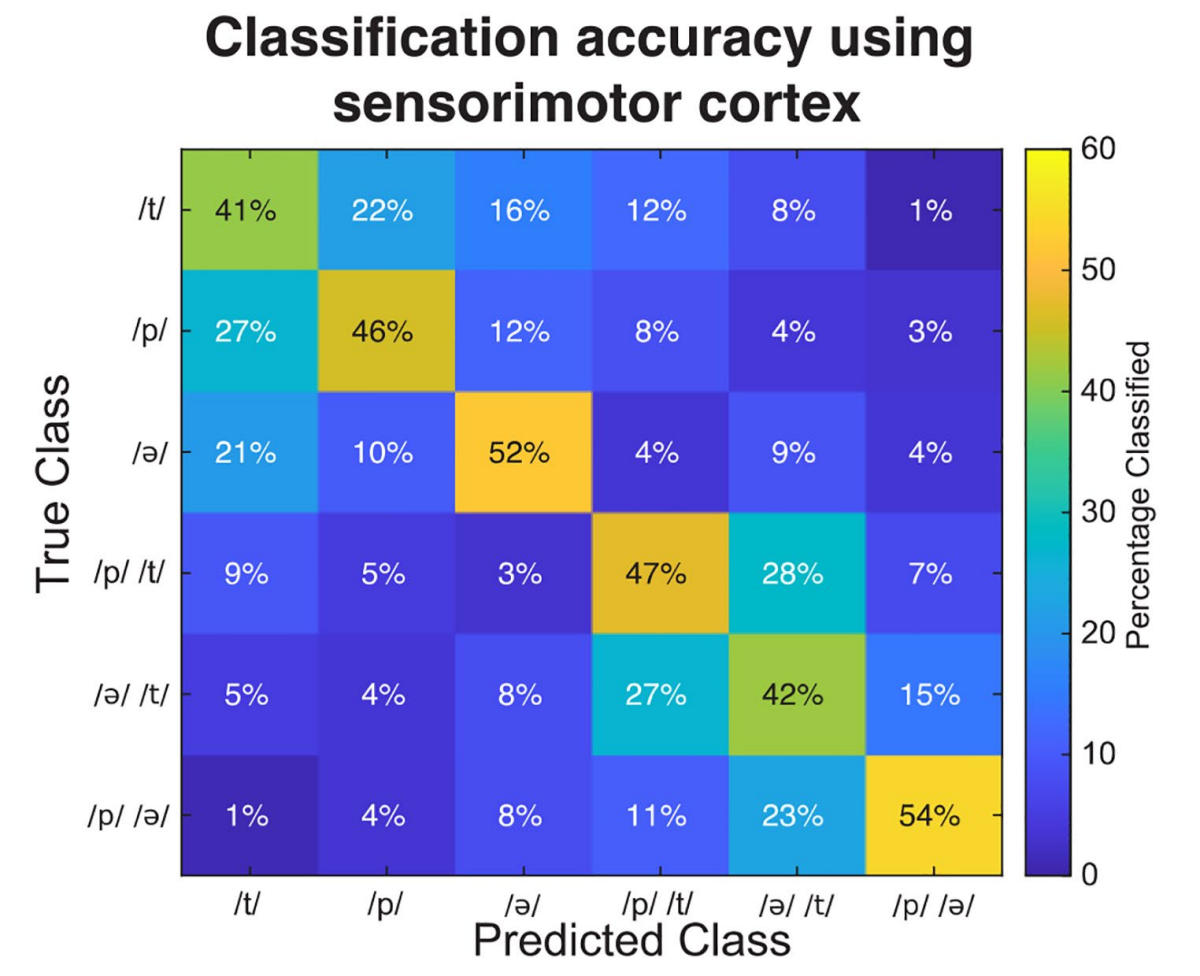
Group mean (*n* = 15) classification accuracy when including all 6 classes for activity in the sensorimotor cortex (blue) and pars triangularis and pars opercularis (green). Adjacent boxes indicate the range of the 25th and 75th percentile, the red line inside the box represents the mean. LR = left and right hemispheres combined; L = left hemisphere; R = right hemisphere. *** *p* <.001, Bonferroni corrected for multiple comparisons

First, we assessed if it was possible to discriminate between all 6 phoneme trial classes. The classification accuracy for the bilateral sensorimotor cortex was significantly above the 16.7% chance level (*a =* 0.05 at a threshold of 22%) for each participant. The mean accuracy across the 15 participants for the bilateral sensorimotor cortex was 43% (SD = 9%; min-max = 33 − 63%; one-sample t_(14)_ = 13.1; *p* <.001; see Table 2; Fig. 3).

**Fig. 4:**
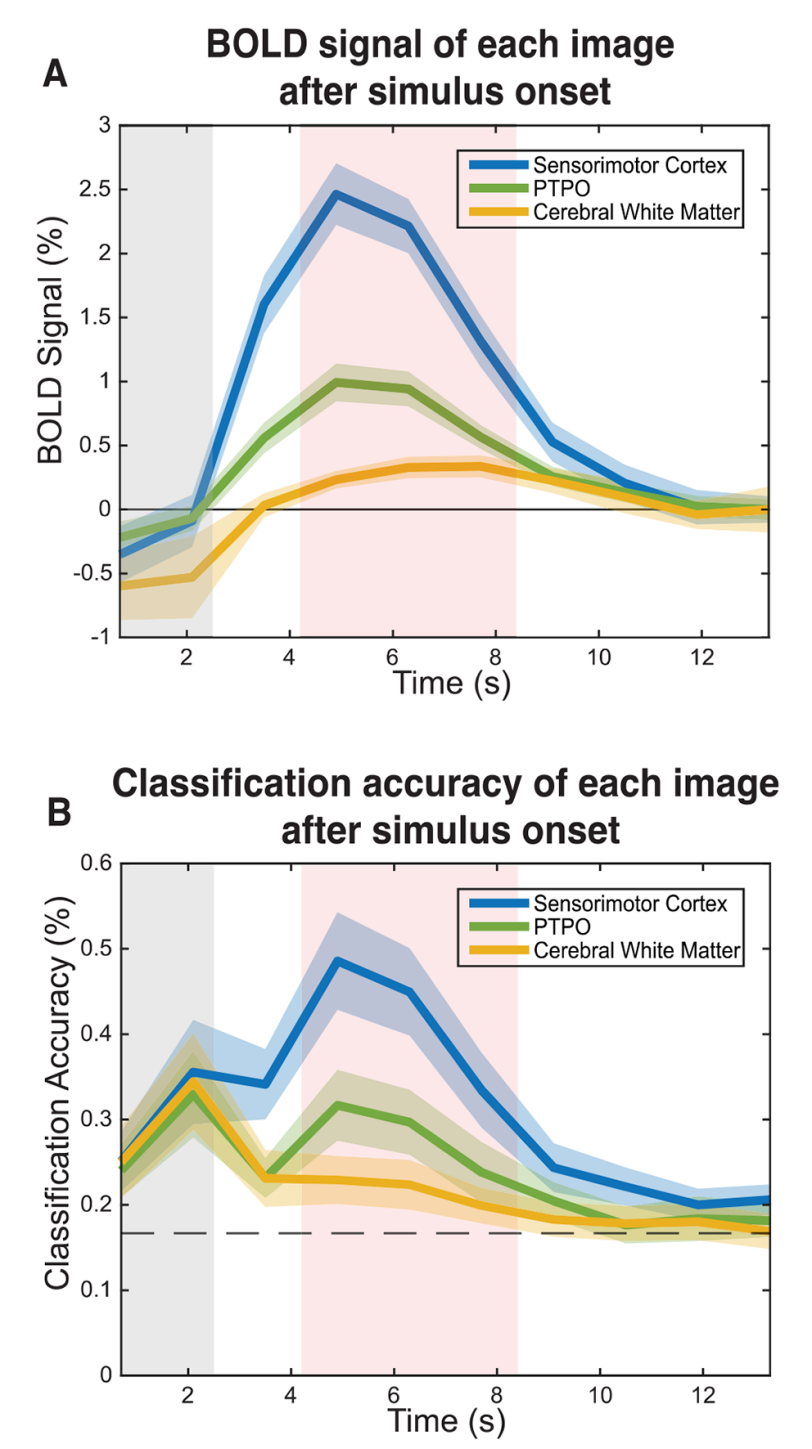
Group mean confusion matrix (*n* = 15) showing classification accuracies for each of the classes based on the activity of the sensorimotor cortex in both hemispheres combined

To investigate the decodability of the distinct classes, we visually inspected the mean confusion matrix across subjects (Fig. 4). The percentage of correctly classified trials for each class ranged from 41 to 54%. We further observed that the decoder confuses significantly more amongst single phoneme trials and amongst paired phoneme trials, instead of confusing single phoneme with paired phoneme trials and vice-versa (paired t_(14)_ = 12.4; *p* <.001).

The classification was repeated using only precentral or postcentral gyrus. The accuracies for the precentral and postcentral gyrus were 45% and 42% respectively (SD = 8.33%; min-max = 29 − 60%; one-sample t_(14)_ = 12.95; *p* <.001 for precentral gyrus; SD = 8.97%; min-max = 29 − 60%; one-sample t_(14)_ = 10.79; *p* <.001 for postcentral gyrus), however no significant difference was observed between the gyri (paired t_(14)_ = 1.64; *p* =.12; Table 2). In addition, we assessed the potential of classifying phonemes based on activity in PTPO. The mean accuracy for PTPO (Table 2; Fig. 3), was 32% (SD = 8%; min-max = 20 − 42%; one-sample t_(14)_ = 7.8; *p* <.001), which was significantly lower than the accuracy obtained for the sensorimotor cortex (Bonferroni corrected paired t_(14)_ = 9.8; *p* <.001).

Visual inspection of the confusion matrix shows that for single phoneme trials, trials with the phoneme /ə/ are more reliably classified (52%) than trials with /t/ and /p/ (41% and 46% respectively). For trials with paired phonemes, the trials with the paired phonemes /p/ /ə/ are more accurately classified (54%) than trials with phonemes /p/ /t/ and /ə/ /t/ (47% and 42% respectively) (Fig. 4). However, Friedman test followed by multiple comparison correction showed the only significant difference was between the conditions /t/ and /p/ /ə/ (mean rank difference = -3.99; adjusted *p* =.02).

### Phoneme Information is Not Lateralized

To assess hemispheric preference for phoneme decoding, we calculated the classification accuracy for the left and right hemisphere separately. This revealed no significant difference between left and right hemispheres for both sensorimotor cortex (paired *t*_(14)_ = 2.3; *p* =.0356, Bonferroni corrected; Table 2; Fig. 3) and PTPO (paired t _(14)_ = 1.7; *p* =.1078, Bonferroni corrected).

Classification when including both hemispheres was significantly higher than for only the right hemisphere, but not only the left hemisphere (Table 2; Fig. 3; both vs. left hemisphere: paired t _(14)_ = 1.1; *p* =.3001, Bonferroni corrected; both vs. right hemisphere: paired t _(14)_ = 4.7; *p* <.001, Bonferroni corrected). Similarly, in the PTPO, the difference between both hemispheres and a left hemisphere was not significant, while the difference between both hemispheres and right hemisphere was significant (Table 2; Fig. 3; both vs. left hemisphere: paired t _(14)_ = 3; *p* =.0091, Bonferroni corrected; both vs. right hemisphere: paired t _(14)_ = 3.6; *p* =.003, Bonferroni corrected).

**Table 2:** Group mean classification performance and standard deviations in percentage accuracy for different areas and hemispheres

		Six classes (chance level 17%)	Single Phoneme Classes (chance level 33%)	Paired Phonemes Classes (chance level 33%)	Training with single testing with paired (chance level 33%)	Training with paired testing with single (chance level 33%)
Sensorimotor Cortex	**Both Hemispheres**	47 ± 9	56 ± 11	57 ± 10	53 ± 9	51 ± 7
	**Left Hemisphere**	45 ± 7	58 ± 10	53 ± 8	51 ± 10	50 ± 8
	**Right Hemisphere**	41 ± 10	52 ± 10	50 ± 8	50 ± 9	46 ± 10
Pars triangularis and pars opercularis	**Both Hemispheres**	32 ± 8	43 ± 10	46 ± 8	44 ± 6	43 ± 6
	**Left Hemisphere**	29 ± 6	40 ± 9	45 ± 8	43 ± 8	42 ± 7
	**Right Hemisphere**	28 ± 7	41 ± 8	40 ± 7	41 ± 5	42 ± 6
Precentral Gyrus	**Both Hemispheres**	45 ± 8	54 ± 10	53 ± 8	52 ± 8	50 ± 6
	**Left Hemisphere**	41 ± 7	52 ± 8	50 ± 8	48 ± 7	46 ± 7
	**Right Hemisphere**	39 ± 9	48 ± 10	46 ± 7	46 ± 8	46 ± 9
Postcentral Gyrus	**Both Hemispheres**	42 ± 9	53 ± 10	51 ± 9	45 ± 10	48 ± 10
	**Left Hemisphere**	40 ± 8	51 ± 7	48 ± 11	46 ± 10	49 ± 8
	**Right Hemisphere**	34 ± 9	49 ± 9	44 ± 8	44 ± 8	43 ± 9
Cerebral White Matter	**Both Hemispheres**	22 ± 5	37 ± 8	37 ± 6	36 ± 5	35 ± 6
	**Left Hemisphere**	21 ± 4	37 ± 7	37 ± 10	35 ± 4	35 ± 6
	**Right Hemisphere**	21 ± 5	35 ± 6	36 ± 8	35 ± 6	36 ± 8

### Functional Images used are free of head Movement Artifacts Associated with Phoneme Production

To inspect whether the observed classification scores were truly based on neural representation, rather than head motion artifacts introduced by articulator movements, we repeated the classification analysis. However, instead of using the mean of voxels across three images as features in the classification procedure, we used the voxel values of each single image after stimulus onset. Effects of head movements on classification scores should only occur during the first 3.5s after trial onset in order to not interfere with results. Classification on images after 4.9s (which corresponds to the 4th image after stimuli onset) should be free of head motion artifacts.

The BOLD signals and classification accuracy at each time point are shown in Fig. 2A and B. In absence of confounding BOLD activity caused by motion artifacts, the classification accuracy over time since stimulus onset should resemble a BOLD-like shape. While evidence for motion artifacts for the images directly following the stimulus may be anticipated due to the presence of mouth movements, this is acceptable as long as there are no indications that they affect the images from which the features were extracted (i.e. images 4, 5, 6).

Accuracy over the image since onset in the sensorimotor cortex and PTPO produced a BOLD like shape, with a peak corresponding to the peak in the BOLD response at 4.9 s, and subsequent decrease until the next stimulus. In contrast to the BOLD responses, there was an increase in the classification accuracy around two seconds after stimulus onset across all ROIs (sensorimotor cortex, PTPO and cerebral white matter). These time points coincide with the period participants were engaging in phoneme production (grey highlighted area in Fig. 2B), and the increased classification accuracy is thus likely driven by motion artifacts. The reduction in BOLD relative to baseline during the first 2 images (Fig. 2A) may indicate that these artefacts operate through imperfect shimming as a result of changes in the position of the articulators during phoneme production relative to rest.

Variations in classification accuracy after 3.5s are similar to the BOLD response, suggesting they are driven by cerebrovascular instead of artifactual signal changes. Hence, it is unlikely that phoneme classification during images 4–6 is related to motion.

### Paired Phonemes can be Decoded by Training the Classifiers with Single Phonemes

**Fig. 5:**
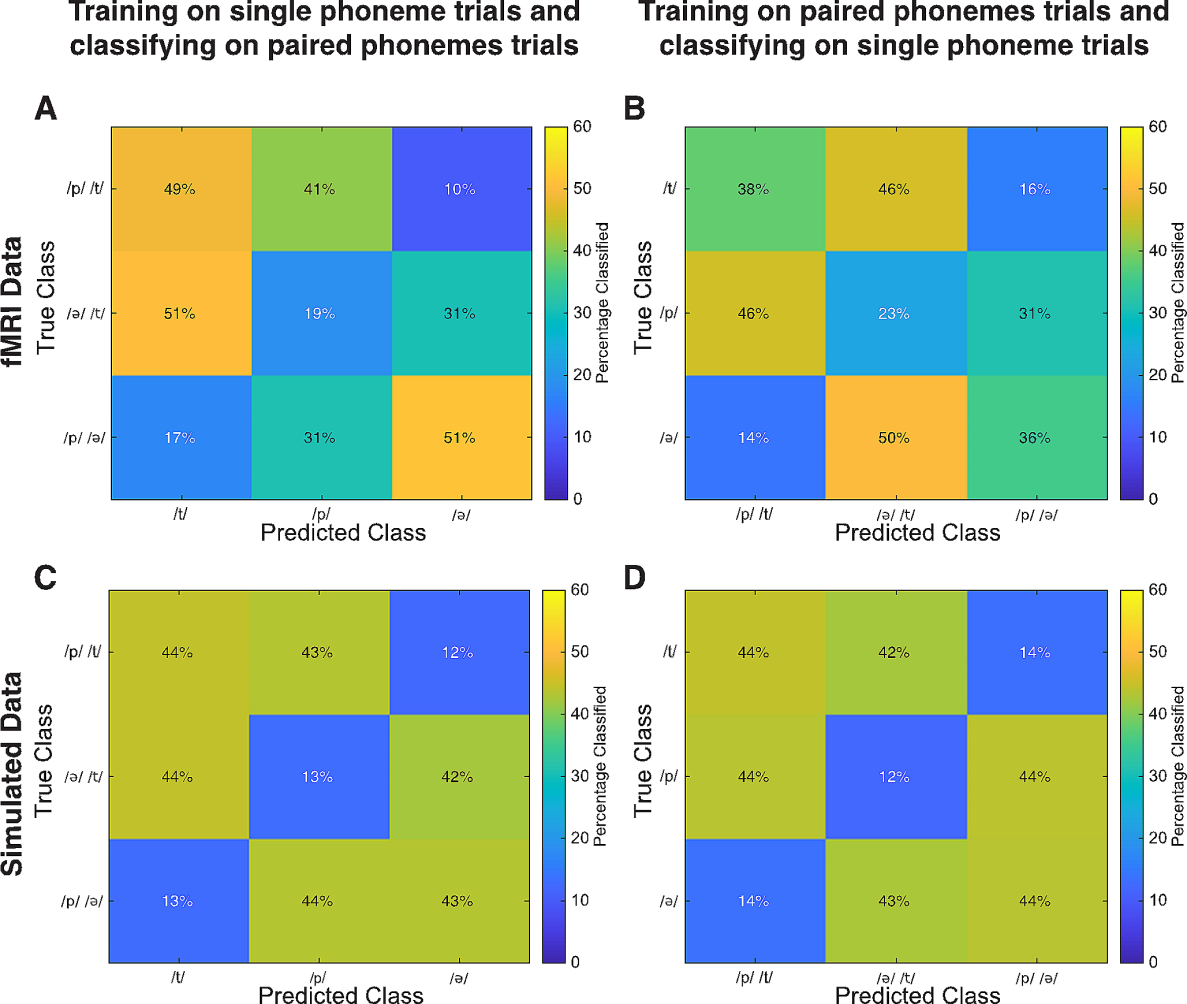
Group-mean classification matrix showing accuracies while training on single phoneme trials and classifying paired phoneme trials (**A** and **C**) and while training on paired phoneme trials and classifying single phoneme trials (**B** and **D**). Panel **A** and **B** represent classification results while using fMRI data from the sensorimotor cortex. Panel **C** and **D** show results when using simulated data, assuming linear addition of single phoneme activity in paired phoneme trials. Note that the matrix is structured so that the cells where the predicted/true single phoneme is absent from the true/predicted paired phoneme trial, are located on the diagonal (bottom-left to upper-right)

To establish the possibility of classifying phoneme combinations based on single phoneme activity, a SVM was trained on activity during production of the single phoneme trials and used to classify paired phoneme trials. The group-mean classification matrix can be seen in Fig. 5A. Results were tested by estimating the accuracy of correctly predicting the absence of a single phoneme in a paired phoneme trial (chance level is 33%). The group-mean accuracy was 53% (SD = 9%; min-max = 38 − 71%; one-sample t_(14)_ = 15.6; *p* <.001) (see Table 2), with 11 out of 15 subjects having a classification accuracy significantly above chance level (*p* <.05). Visual inspection of the confusion matrix revealed that when classifiers were trained on single phonemes and tested on paired phoneme trials, the group-mean classification matrix reveals a highest accuracy for the detection of the second phoneme produced, however this difference was not significant (paired t_(14)_ = 0.24; *p* =.81). Results of the reversed classification scheme, where isolated phoneme trials are predicted based on the activity of paired phonemes trials, were significant as well (Group-mean accuracy = 51%; SD = 7%; min-max = 39 − 61%; one-sample t_(14)_ = 20.1; *p* <.001) (see Table 2; Fig. 5B). Importantly, the accuracy of predicting the phonemes present in the conditions was significantly higher than the accuracy of predicting the absent phonemes for both schemes (paired t_(14)_ = -10.33; *p* <.001 for training on single phonemes and testing on paired; paired t_(14)_ = -9.69; *p* <.001 for training on paired and testing on single phonemes).

### Classification of Simulated data that Assumes Linear Addition of Phoneme Activity is Similar to fMRI data

To verify that obtained classification results were consistent with paired phonemes being linear additions of single phonemes, we performed an additional analysis based on simulated data. Simulated data, meant to represent the extracted BOLD signal of the 3 individual phonemes, was generated by creating 3 fixed normally distributed patterns of 1000 features. Independent normally distributed noise was added to the pattern of every individual trial. The signal to noise ratio was adjusted until the classification accuracy while using simulated data representing the isolated phoneme trials was similar to the classification accuracy obtained to classify individual phonemes with BOLD signal (chance level of 33%). In order to simulate the combined phonemes trials, the patterns representing 2 of the isolated phonemes were linearly combined. The decodability of linearly combined simulated data was assessed by training SVM with simulated isolated phoneme trials and used to classify simulated combined trials, and vice-versa. Data was randomly generated one hundred times, and classification accuracies and confusion matrices were averaged across one hundred repetitions. The pattern of results was similar to those obtained with the real data (see Fig. 5C and D). Note that these results indicate consistency with linear addition of phoneme activity, but that other underlying mechanisms might produce similar results.

### Local Information to Decode Phonemes is Localized around the Central Sulcus

**Fig. 6:**
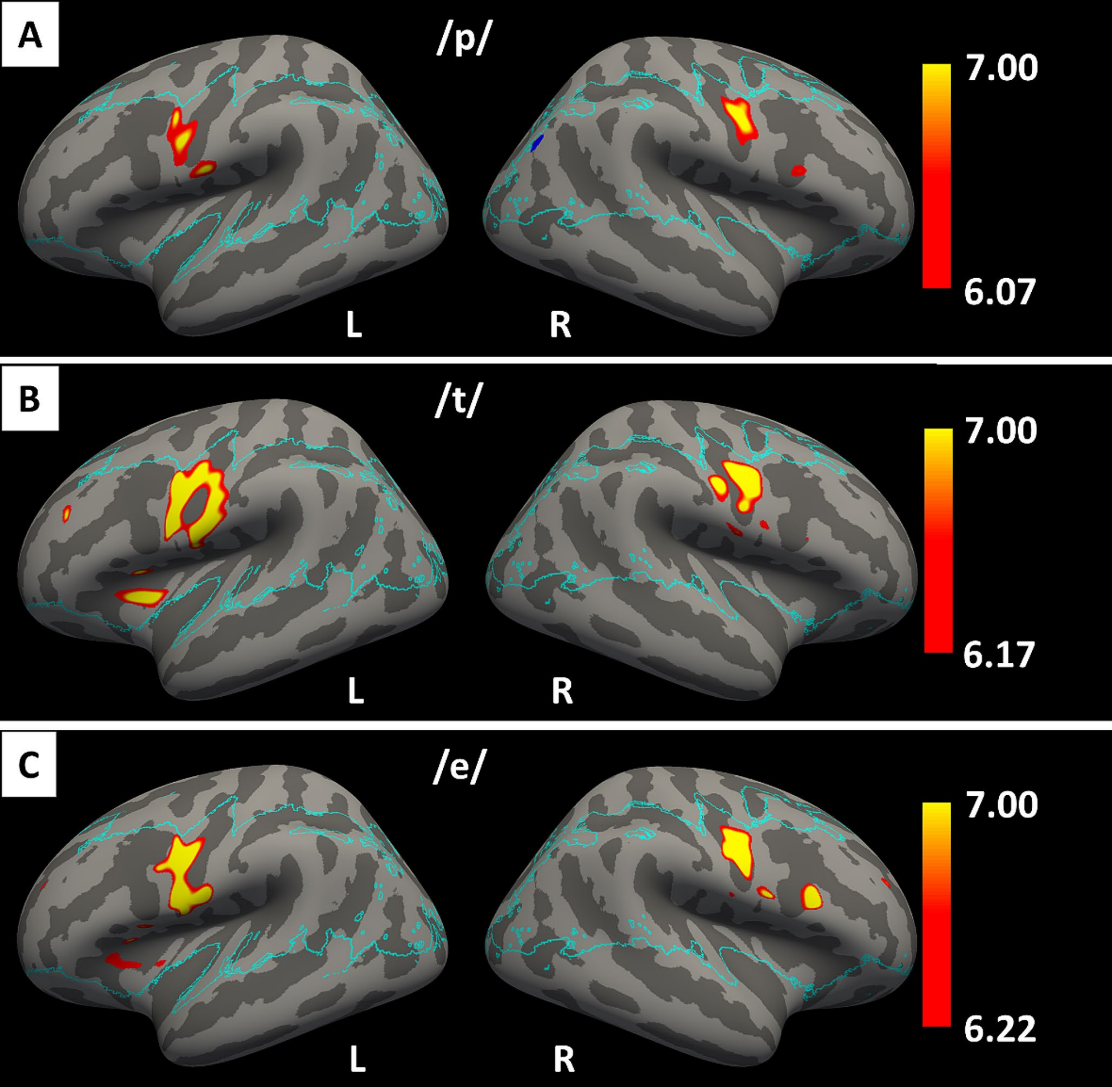
Results of the univariate analysis comparing activity with rest on an inflated surface for the /p/ phoneme (**A**), the /t/ phoneme (**B**), and the /ə/ phoneme (**C**). The blue line marks the border of the area that was included in the groupwise analysis. Multiple comparisons correction with family wise error correction based on Random Field Theory (*p* <.05)

**Fig. 7:**
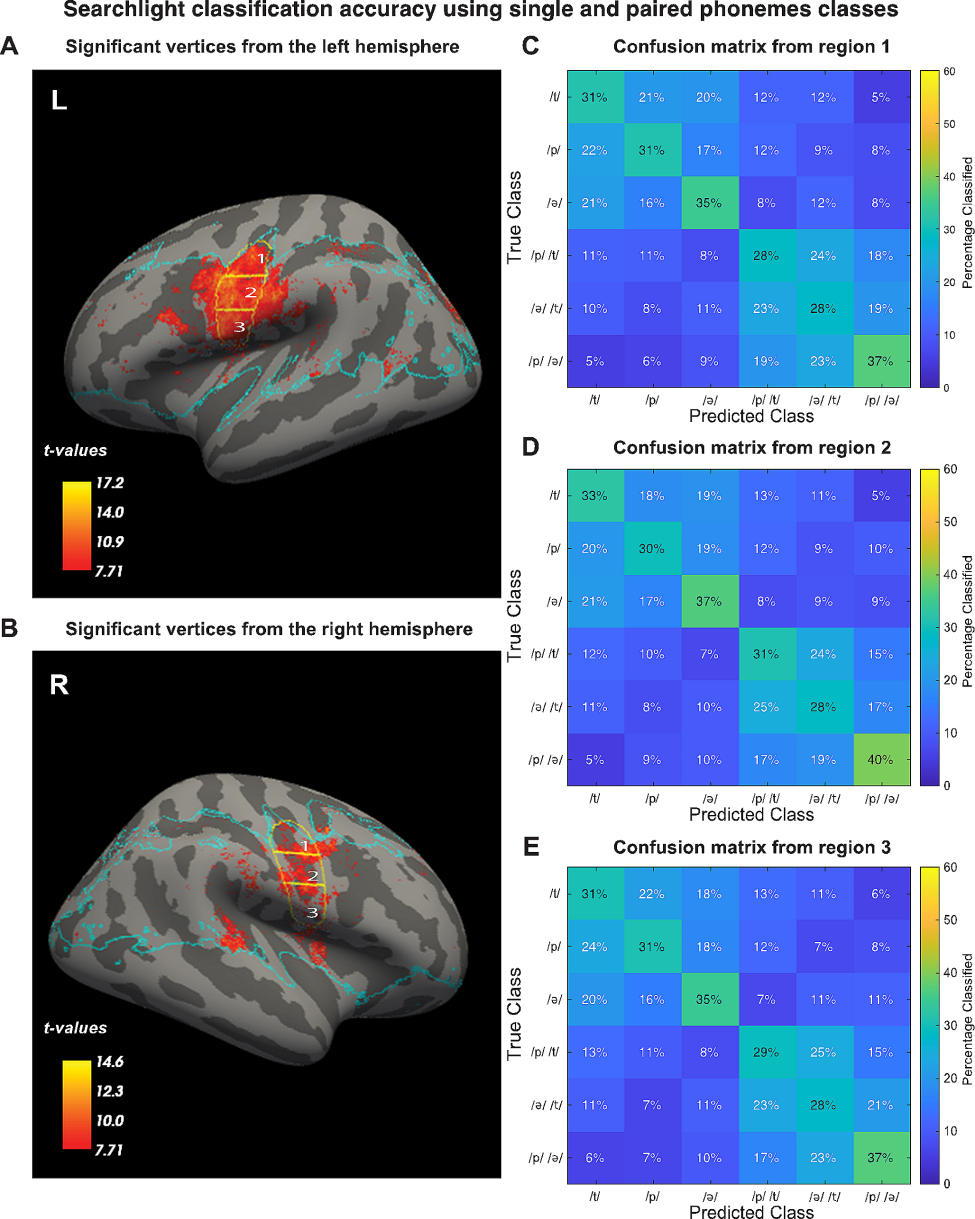
Significant groupwise (*n* = 15) searchlight classification results while including all classes, superimposed on the inflated surface template of the left (**A**) and right (**B**) hemisphere. The panels on the right show the mean confusion matrices for significant (*p* <.05) vertices in the superior (**C**), medial (**D**) and inferior (**E**) part of the sensorimotor cortex. The blue line marks the border of the area that was included in the groupwise analysis. Multiple comparisons correction with family wise error correction based on Random Field Theory (*p* <.05)

Univariate and a searchlight SVM approaches were used to obtain more insight in the spatial distribution that is underlying phoneme activation and information used for classification. Univariate results show that there was significant activity when comparing each single phoneme condition with rest in the ventral sensorimotor cortex bilaterally (Fig. 6). However, for none of the comparisons between single phoneme conditions were there any voxels with significantly different activity (not shown). The groupwise searchlight-results including all 6 classes show that most relevant discriminability is in the mouth area of the ventral sensorimotor cortex (Fig. 7A and B). In order to investigate the nature of the distribution in more detail, three ROIs were manually defined that covered the superior, medial, and inferior part of the ventral sensorimotor cortex. Visual inspection of the mean confusion matrices in these ROIs (Fig. 7C, D, E), indicated that significant accuracy was primarily driven by classification between single and paired phoneme trials.

**Fig. 8:**
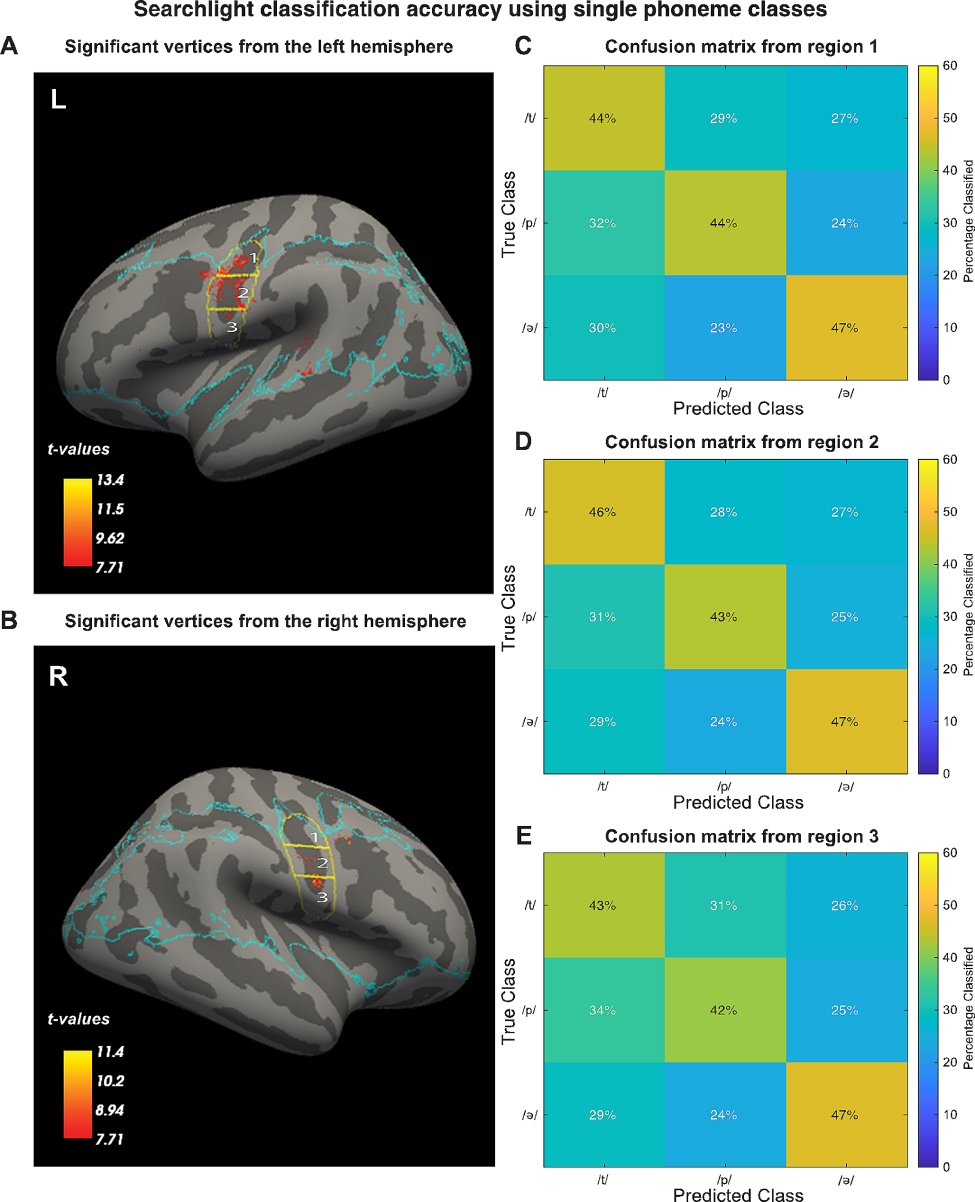
Significant groupwise (*n* = 15) searchlight classification results while including only single phoneme trials, superimposed on the inflated surface template of the left (**A**) and right (**B**) hemisphere. The panels on the right show the mean confusion matrices for significant (*p* <.05) vertices in the superior (**C**), medial (**D**) and inferior (**E**) part of the sensorimotor cortex. The blue line marks the border of the area that was included in the groupwise analysis. Multiple comparisons correction with family wise error correction based on Random Field Theory (*p* <.05)

Furthermore, we repeated the searchlight procedure with including only single phoneme trials. This analysis revealed far fewer significant vertices, but most were still located in the ventral sensorimotor cortex (Fig. 8A and B). In order to assess if the searchlight results included evidence that discriminability was based on differential engagement of the somatotopic representations of the main articulators for each single phoneme class, the same three ROIs (superior, medial, and inferior) were used to calculate mean confusion matrices. According to this rationale, e.g. the lip area (superior ROI) may accurately identify a /p/ phoneme, while confusing the other phonemes. The mean confusion matrices for different portions of the ventral sensorimotor cortex did however not vary substantially, suggesting that classification between single phonemes is not driven by differential somatotopic activity (see Fig. 8C, D and E).

## Discussion

In this study, we demonstrated the possibility of classifying phonemes pronounced in isolation and in pairs based on BOLD activity in the sensorimotor cortex and PTPO, with sensorimotor cortex achieving the highest accuracy (see Table 2; Fig. 3). Additionally, we were able to classify phonemes that were pronounced in pairs while using support vector machines trained on isolated phonemes (see Table 2; Fig. 5A), indicating the activity patterns of single phonemes is recognizable when phonemes are pronounced in combinations. Simulation results indicate this is in line with the activity of combined phonemes being linear combinations of individual phonemes. However, more detailed analyses using searchlight support vector machines did not reveal evidence that this was due to a straightforward somatotopic addition of activity of individual articulators. Our results are of importance for creating a speech BCI that works by detecting the presence of individual phonemes in spoken words.

Our results for the sensorimotor cortex are in line with previous fMRI experiments investigating the feasibility of classifying mouth-actuator-activity. Bleichner et al. used 7T-fMRI to classify 4 different mouth movements with an 80% accuracy (Bleichner et al. 2015), and Correia et al. achieved significant classification based on BOLD responses for voiced and whispered phonatory gestures involving the tongue, lips and velum (Correia et al. 2020). Results from ECoG measurements further confirm the sensorimotor cortex as a suitable area for decoding speech-related brain activity. This includes the classification of phonemes (Ramsey et al. 2018), vowels and consonants from consonant-vowel syllables (Livezey et al. 2019), letter sequences using code words (Metzger et al. 2022) and sentences (Moses et al. 2021). Additionally, there was no significant difference between the classification accuracy of the precentral and postcentral gyrus. Previous studies have demonstrated that somatosensory representations are still present for patients with limited or no motor output and amputees (Bruurmijn et al. 2017), suggesting that they may also be preserved after paralysis and thus provide a target for BCIs.

Importantly, the main novel finding in our study is the establishment of the feasibility of detecting single phonemes could be detected when pronounced in pairs, when training on single phoneme activity. This finding establishes a proof of principle for detecting the building blocks of language production, allowing machine learning on single phonemes to classify words. This is in line with previous ECoG studies that demonstrated the feasibility of discriminating phonemes within words (Mugler et al. 2014a, b). Note however that the paired phonemes in our study were pronounced consecutively with 1 s interval, which is not representative for word pronunciation during natural language production. E.g., Salari et al. demonstrated that neural signatures are influenced by previous speech movements when spaced a second or less apart using repeated vowel production (Salari et al. 2019) and that including neural patterns of a vowel pronounced in different contexts (isolation or preceded by other vowel) in the training set performs better than training the classifier for each context (Salari et al. 2018). The influence of these complicating interactions seems to be limited at the pace of phoneme production that we chose, as the results we observe are roughly similar to those acquired when classifying based on simulated data where paired-phoneme trial data was created by linear addition of the activity of single phonemes. A speech BCI using the principles applied in this experiment could thus be feasible up to a speed of at least 1 phoneme per second.

Using a searchlight SVM, we found that phoneme-classification based on local information was possible primarily in the ventral sensorimotor cortex, particularly close to the central sulcus (Fig. 6A and B). However, the confusion matrices of the significant vertices indicated that the classifiers were mostly differentiating between single and paired phonemes (Fig. 6C, D and E). It is thus most likely that significant local classification is caused simply by trials with paired phonemes yielding higher activity than trials with single phonemes, since increasing the quantity of stimulation should enhance BOLD responses. To account for this, the searchlight analysis was repeated using single phoneme trials only (Fig. 7A and B). While this analysis revealed a few locations in the ventral sensorimotor cortex where significant classification based on local information was possible, the extent of these areas was far less than when including all classes. In addition, the confusion matrices for different portions of the ventral sensorimotor cortex were highly similar, indicating that pronunciation of the different phonemes did not result in mapping to the Penfield representations due to differential use of tongue, or larynx due articulators (Figure C, D and E). Also, the univariate comparisons between the single phoneme conditions did not reveal any significant differences. The absence of clearly localized patterns is in line with more variable articulator representation than has been reported previously (Carey et al. 2017). Studies with ECoG have also reported more broadly distributed and overlapping combinations of electrodes to decode phonemes and syllables (Ramsey et al. 2018; Bouchard et al. 2013; Conant et al. 2014). These results suggest that for the purpose of a speech-BCI it would be beneficial to cover a more extensive part of the ventral sensorimotor cortex.

Despite the fact that most BCIs have focused on neural signals originating from the sensorimotor cortex because of its somatotopic organization, other brain regions may be suitable for BCI purposes as well (Gallego et al. 2022). Broca’s area has been shown to have a roles in speech production, ranging from representation of articulatory programs to higher linguistic mechanisms (Papitto et al. 2020; Fedorenko and Blank 2020). However, it has received far less attention in speech decoding studies than the sensorimotor cortex, especially among studies using intracranial recordings. The classification accuracy from PTPO was significant, but lower than for the sensorimotor area (Table 2; Fig. 3), suggesting PTPO to be sub-optimal for a BCI implant. These results align with result those of previous studies that showed no electrodes significantly decoded covertly produced vowels (Ikeda et al. 2014), no activity recorded during articulation of single words using ECoG (Flinker et al. 2015) and no speech information during attempted speech using microarrays (Willett et al. 2023). On the other hand, there is evidence for the possibility to decode overtly and covertly produced vowels and consonants within phonological sequences (Pei et al. 2011). Results of studies so far that compared neuronal representations of overt, covert and attempted speech observed substantial overlap (Palmer et al. 2001; Shuster and Lemieux 2005; Zhang et al. 2020; Brumberg et al. 2016; Soroush et al. 2023) and classifiers trained on attempted speech have been successfully used to classify overt speech based on features acquired using intracranial recordings (Metzger et al. 2023; Willett et al. 2023). Additionally, it was shown that Broca’s neural activity predicted speech onset in other intracranial studies (Delfino et al. 2021; Castellucci et al. 2022; Rao et al. 2017). This evidence is further supplemented by recent establishment of Broca’s involvement in higher-level language processes such as sequencing, syntax, lexical selection, among others (Bohland and Guenther 2006; Riecker et al. 2008; Conner et al. 2019; Matchin and Hickok 2020; Deldar et al. 2020). The task used in our study most likely did not sufficiently engage these speech related functions.

Searchlight results using all phoneme classes revealed significant classification in the temporal lobe. As participants overtly pronounced the phonemes, this could have been the result of responses in the primary auditory cortices. Whether this did in fact occur is unclear, as subjects wore hearing protection and were in a noisy environment. Nevertheless, auditory stimulation could have happened through bone conduction. If classification from auditory cortex in our subjects is based on acoustic stimulation, it would not be feasible in locked-in patients, given that they are expected to produce attempted speech with limited or no auditory output. However, some studies observed activation of auditory regions in covert, imagined and attempted speech (Metzger et al. 2023; Zhang et al. 2020; Soroush et al. 2023; Martin et al. 2016), but it needs to be further explored whether this can be used for distinguishing words. In addition, as our primary focus was on the sensorimotor cortex, we defined the field of view to maximize voxel coverage within this area. Consequently, not all subjects achieved complete coverage of the auditory regions in the temporal lobe.

Our results did not show a difference in phoneme classification between the left and right hemisphere in both sensorimotor cortex and PTPO (Table 2; Fig. 3), indicating both hemispheres are a suitable location for a speech-BCI implant. Studies on motor planning of speech production have observed left lateralization in inferior frontal gyrus and ventral premotor cortex (Riecker et al. 2005; Peeva et al. 2010; Ghosh et al. 2008; Kearney and Guenther 2019). Additionally, our results do not show a hemispheric preference for classification based on activity in PTPO, although this might be caused by the aforementioned lack of syntactical and lexical processing involved in the task that we used. The results we obtained for the sensorimotor cortex are however in line with several studies that found bilateral activation in this region during speech related movements (Ramsey et al. 2018).

Investigating articulators using fMRI comes with the challenge of motion artifacts. Displacement of the articulators affects functional images that are acquired during phoneme production, thereby confounding the measured BOLD responses. To account for this, we only included features from functional images acquired after the phoneme production, which were shown to be unaffected by artifacts (Fig. 2). Unfortunately, such speech-related artifacts are likely to limit the interpretability of BOLD signals acquired during continuous speech, imposing some limits on fMRI research of overt language production.

A limitation of the current experimental design is that it is based on overt speech production, while the target population for a speech BCI is in a locked in state, and consequently not able to produce overt speech. However, ECoG grids on the sensorimotor cortex that decode attempted hand movements have proven to be feasible (Pandarinath et al. 2017; Willett et al. 2021; Vansteensel et al. 2016; Hochberg et al. 2012). Moreover, there is a correlation between sensorimotor patterns of actual and attempted hand movements (Bruurmijn et al. 2017). One would assume the same to apply to activity patterns in sensorimotor cortex during articulator movements. Nevertheless, a necessary next step involves the assessment of replicability of the current findings in locked in patients.

While fMRI at 3T has repeatedly been shown to provide insightful information regarding speech dynamics (Correia et al. 2020; Grabski et al. 2012), high-field fMRI allows measurements at increased spatial resolutions while maintaining adequate signal to noise ratios (Formisano and Kriegeskorte 2012). Therefore, it is expected that fMRI at 7T provides details in the activity patterns that are not accessible at 3T (Formisano and Kriegeskorte 2012; Chaimow et al. 2018), despite the fact 7T is more susceptible to distortions and artifacts. However, the current design using fMRI is not fully representative for the actual BCI, that would most likely be based on electrophysiological measurements. While 7T fMRI has substantially contributed to findings regarding brain activity related to speech production, and has shown to correlate with ECoG in the sensorimotor cortex (Siero et al. 2013, 2014), the characteristics of hemodynamic responses inherently limit its temporal resolution. We were therefore only able to access spatial features of brain activity related to phoneme production, and effectively ignoring variations in brain activity during the complex sequences of articulator movements. Such features could of course be used in a BCI based electrophysiological measurements. In addition, the necessity to use a slow event related fMRI design limited the total number of trials to train the classifiers. Such limitation would not apply to a BCI that is based on electrophysiology, allowing a far greater number of trials to train the classifiers, and thus increase its performance.

In conclusion, we showed that it is possible to decode individual phonemes and paired phonemes that were pronounced overtly while acquiring the BOLD signal in the sensorimotor cortex. Notably, by demonstrating the possibility of classification of paired phonemes while training the classifier on single phonemes, we provided a proof of concept of a BCI that detects phonemes present in speech. Future research is needed to assess the detection of phonemes present spoken words to further explore the feasibility of a phoneme based BCI using invasive electrophysiology.

## Data Availability

Raw MRI data will be available upon request to the authors. MRI data is considered personal data pursuant to General data protection regulation (GDPR) and can only be shared based on and subject to the Royal Netherlands Academy of Arts and Sciences (KNAW) policies. Considering the requirements imposed by law and the sensitive nature of personal data, any requests will be addressed on a case-by-case basis, subject to a data usage agreement.
